# Novel Insights into the Effects of Genetic Variants on Serum Urate Response to an Acute Fructose Challenge: A Pilot Study

**DOI:** 10.3390/nu14194030

**Published:** 2022-09-28

**Authors:** Xinruo Zhang, Baba B. Mass, Valentina Talevi, Ruixue Hou, Kari E. North, Venkata Saroja Voruganti

**Affiliations:** 1 Department of Nutrition and Nutrition Research Institute, University of North Carolina at Chapel Hill, Kannapolis, NC 28081, USA; 2 Department of Epidemiology, Gillings School of Global Public Health, University of North Carolina at Chapel Hill, Chapel Hill, NC 27599, USA; 3 Department of Population Health Science and Policy, Icahn School of Medicine at Mount Sinai, New York, NY 10029, USA

**Keywords:** nutrient challenge, sugar-sweetened beverages, hyperuricemia, single nucleotide polymorphism (SNP)

## Abstract

Studies have shown that genetic variations can influence metabolic response to nutrient intake, and that diets rich in fructose contribute to hyperuricemia. In this pilot study, our aim was to determine the variability of serum urate in response to an acute fructose challenge and to investigate if genetic variants would affect this response in young to middle-aged adults who self-reported as Black or White. Fifty-seven participants consumed a fructose-rich beverage after an overnight fast. Blood was drawn at five time points (baseline, 30, 60, 120, and 180 min after consumption). Thirty urate-related single nucleotide polymorphisms (SNPs) were analyzed for their associations with baseline serum urate and its percent changes, using a two-step modeling approach followed by meta-analysis. At baseline, serum urate (mg/dL, mean ± SD) was higher in Whites (5.60 ± 1.01 vs. 5.37 ± 0.96), men (6.17 ± 1.14 vs. 5.24 ± 0.79), and those with obesity (5.69 ± 1.08 vs. 5.42 ± 1.06 vs. 5.34 ± 0.80). Three SNPs were significantly associated with baseline serum urate or its percent changes, and six SNPs were nominally associated with percent changes in serum urate. In summary, our results showed that genetic variants could play a role in short-term urate metabolism.

## 1. Introduction

Circulating in the bloodstream as urate anion, uric acid is the end product of purine metabolism. Hyperuricemia develops when serum urate concentration exceeds the limit of solubility, 6.8 mg/dL [1]. Hyperuricemia is a risk factor for gout, a painful form of arthritis, and it increases the risk for metabolic diseases such as cardiovascular disease, type 2 diabetes, and chronic kidney disease [2]. It was estimated that in 2015 to 2016, 47 million adults had hyperuricemia, while 9 million adults were living with gout in the United States [3].

Both environment and genetics affect one’s serum urate concentrations. Dietary factors such as fructose have been implicated in the development of hyperuricemia [1]. Fructose, a monosaccharide that occurs naturally in fruits and vegetables, is also a common added sugar found in processed foods or sugar-sweetened beverages (SSB). Although the consumption of added sugar has been declining since 2013, its absolute consumption remained above the recommended intake level [4]. After consuming fructose in large quantity, adenosine triphosphate (ATP) quickly exhausts to supply the phosphate needed in fructose metabolism in an unregulated fashion; the resulting adenosine monophosphate (AMP) is then converted into uric acid by a series of enzymatic reactions [5]. Indeed, past intervention studies have corroborated the urate-raising effect of fructose [6,7,8,9,10,11,12,13]. 

On the other hand, using cross-sectional urate measurements, genome-wide association studies (GWAS) have identified several single nucleotide polymorphisms (SNP) associated with urate, most of which are common variants with modest effects [14,15]. One’s blood urate can vary significantly throughout the day or from day to day, in response to changes in the environment and physiological state. Still, to date, only a handful studies have examined the roles of genetic variants in serum urate response to a dietary challenge [8,9,10], and such studies can offer valuable insights into the short-term roles genetic variants play in urate metabolism. In this pilot study, 57 participants were recruited to consume a SSB containing 80% fructose after overnight fasting. Their serum urate concentrations were measured five times within three hours of consumption. Our aims were (1) to determine if there were variations in serum urate after the fructose challenge, (2) to investigate if genetic variants were associated with percent changes in serum urate, and (3) to determine whether factors such as sex and race influenced changes in serum urate concentrations. 

## 2. Materials and Methods

### 2.1. Study Participants

The Fructose Challenge Study (FCS) is a pilot and feasibility study with a dietary intervention designed to investigate the acute effect of a SSB containing 80% fructose on serum urate in adults of two racial groups—Black and White. The FCS was carried out at the University of North Carolina at Chapel Hill Nutrition Research Institute (NRI) in Kannapolis, North Carolina. Specifically, men and women were recruited by website advertisements and fliers posted in the Kannapolis area between September 2016 and February 2017. Inclusion criteria were 30 to 50 years of age and self-identification as Black or White. Race and sex were both self-reported; specifically, staff members asked participants in an open-ended fashion, “what is your race and ethnicity”. Self-reported sex was later confirmed to be consistent with genetic sex. Exclusion criteria included the following self-reported conditions: diabetes, chronic kidney disease, and fructose intolerance. The protocol (16-0876) was approved by the Institutional Review Board of University of North Carolina at Chapel Hill. All participants provided written informed consent before their inclusion.

### 2.2. Study Design

**Figure 1:**
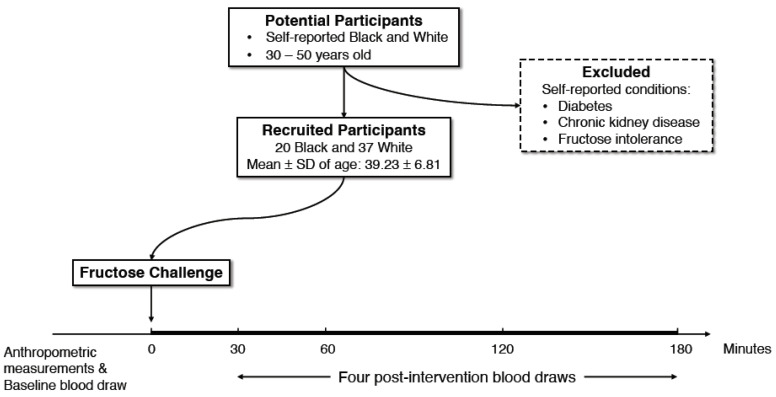
Study design. The Fructose Challenge Study (FCS) is a pilot study with a dietary intervention designed to investigate the acute effects of a sugar-sweetened beverage (SSB) containing 80% fructose on serum urate concentrations in adults 30–50 years of age who self-identified as Black or White.

Following a 12-h overnight fast, participants were invited to the NRI between 06:45 and 08:00 a.m. They were asked to refrain from consuming alcoholic beverages the night before the study. Study design is illustrated using a flow chart in Figure 1. 

First, anthropometrics were measured and recorded. Body composition was assessed by bioelectric impedance analysis (BIA) using Tanita Dual Frequency Total Body Composition Analyzer (DC-430U, Tokyo, Japan). Weight measurements were conducted in a standing position, with subjects wearing light clothing and without shoes. Height was measured to the nearest 0.1 cm in an upright standing position by a stadiometer situated against the wall. Weight and height were then used to calculate body mass index (BMI) as kg/m^2^. Standard definitions were used to categorize BMI as underweight (BMI < 18.5 kg/m^2^), healthy weight (BMI 18.5–24.9 kg/m^2^), overweight (BMI 25.0–29.9 kg/m^2^), or obese (BMI ≥ 30 kg/m^2^). Waist circumference (WC) was measured using a stretch-resistant tape at the midpoint between lower margin of the least rib and the top of the iliac crest to the nearest 0.1 inch. Waist-to-height ratio (WHtR) was calculated by dividing WC (inch) by height (inch). All the anthropometric measurements were taken by the same staff member to minimize measurement variation and margin of error. Blood pressure (BP) was measured using an Omron digital blood pressure monitor (HEM907XL, Omron Healthcare Inc., Lake Forest, IL, USA). Two measurements of BP were taken from the right arm with an interval of 1 min; the average was calculated and used in the statistical analysis. 

Next, participants were instructed to consume a SSB within 15 min. The drink was modeled on an oral glucose tolerance test (OGTT) containing 75 g of glucose and was prepared by dissolving 60 g of fructose (Now Foods, Bloomingdale, IL, USA) and 15 g of glucose (Now Foods, Bloomingdale, IL, USA) in 300 mL of water, equivalent to a concentration of 200 g fructose/L. Each SSB provided 300 kcal. This ratio of fructose to glucose (80:20) was determined based on Akhavan and Anderson’s finding [6]. They reported that the 80:20 ratio elicited the highest urate response after examining the effect of various combinations of fructose and glucose on urate and other metabolic variables. The addition of glucose was to alleviate symptoms of gastrointestinal discomfort caused [16].

### 2.3. Sample Collection and Blood Processing

Blood was drawn before the SSB consumption and 30, 60, 120, and 180 min after the consumption. A trained phlebotomist collected blood through venous puncture using 6-mL ethylenediaminetetraacetic acid (EDTA)-coated tubes and serum tubes (BD Vacutainer, Becton, Dickinson & Company, Franklin Lakes, NJ, USA). Within two hours collection, EDTA tubes were placed on wet ice and centrifuged at 3000 RPM for 15 min at 4 °C. Serum, plasma, and buffy coat were aliquoted and stored at −80 °C. 

### 2.4. Measurements of Serum Urate

At each of the five time points, concentrations of serum urate were measured using fluorometric assays, according to the manufacturer’s instructions (Sigma-Aldrich, St. Louis, MO, USA) on a BioTek Synergy 2 Multi-Mode plate reader (BioTek, Winooski, VT, USA). All samples were analyzed in duplicates and coefficient of variation was <5%.

### 2.5. DNA Extraction, SNP Selection and Genotyping

DNA was extracted from buffy coat using QIAamp DNA Blood Mini Kit (Qiagen Sciences, Valencia, CA, USA). Concentration of genomic DNA was measured using NanoDrop Spectrophotometer. A total of 30 SNPs were selected based on their involvement in or association with fructose metabolism [Solute carrier family 2, member 9 (*SLC2A9*), Protein kinase AMP-activated non-catalytic subunit gamma 2 (*PRKAG2*), Insulin receptor (*INSR*)], urate transport [Leucine rich repeat containing 16A (*LRRC16A*), Solute carrier family 2, member 9 (*SLC2A9*), Solute carrier family 16, member 9 (*SLC16A9*), Solute carrier family 17, member 1 (*SLC17A1*), Solute carrier family 17, member 3 (*SLC17A3*), Solute carrier family 22, member 7 (*SLC22A7*), uromodulin (*UMOD*), Insulin receptor (*INSR*), ATP-binding cassette, subfamily G, member 2 (*ABCG2*)]; and published reports of associations with serum urate [Tripartite motif containing 46 (*TRIM46*), Inhibin subunit beta B *(INHBB*), Inhibin subunit beta C (*INHBC*), Origin recognition complex subunit 4 (*ORC4*), Bromodomain adjacent to zinc finger domain 1B (*BAZ1B),* Membrane bound O-acyltransferase domain containing 4 (*MBOAT4*), Hepatocyte nuclear factor 4 gamma (*HNF4G*), APOBEC1 complementation factors (*A1CF*), RAS like estrogen regulated growth inhibitor (*RERG*), Neurexin 2 (*NRXN2*), Activin A receptor type 1B (*ACVR1B*), insulin-like growth factor 1 receptor (*IGF1R*), UDP-GlcNAc:betaGal beta-1,3 *N*-Acetylglucosaminyltransferase 4 (*B3GNT4*), HLF transcription factor, PAR bZIP family member (*HLF*), Glutamine rich 2 (*QRICH2*)] [14,17,18].

Twenty-seven SNPs were genotyped as part of a genotyping array, Mega8 (Illumina Inc., San Diego, CA, USA). SNPs that were not available in the array were genotyped separately, as follows. SNP rs2231142 was genotyped using TaqMan SNP Drug Metabolism Genotyping Assay (Applied Biosystems, Foster City, CA, USA). In each reaction well, 10 ng of genomic DNA was mixed with 2X TaqMan Universal PCR Master Mix and 20X Drug Metabolism Genotyping Array Mix, according to the manufacturer’s instructions. Real-time PCR was then performed using Eppendorf RealPlex 2 Mastercycler, under the thermal cycling conditions of initial hold (95 °C, 10 min), followed by 50 cycles of denaturation (92 °C, 15 s) and annealing/extension (60 °C, 90 s). Two SNPs, rs16890979 and rs1183201, were genotyped using TaqMan predesigned SNP genotyping assay (Applied Biosystems, Foster City, CA, USA). Each genomic DNA sample (10 ng) was amplified with 2X TaqMan Universal PCR Master Mix and corresponding 20X TaqMan SNP genotyping assay. Real-time PCR was performed using Eppendorf RealPlex 2 Mastercycler, under the thermal cycling conditions of initial hold (95 °C, 10 min), followed by 50 cycles of denaturation (92 °C, 15 s) and annealing/extension (60 °C, 60 s).

### 2.6. Statistical Analysis

Baseline characteristics are presented as mean and standard deviation for the full cohort and by sex in Table 1; each variable was compared between sex using the Welch’s *t*-test. All the baseline variables in Table 1 were complete in all 57 participants. Two of the SNPs (rs16890979 and rs1183201) were genotyped in all participants, while the remaining 28 SNPs were genotyped in 56 participants.

The study outcomes are measurements of baseline serum urate and the four percent changes in serum urate between two adjacent time points (0–30, 30–60, 60–120, and 120–180 min). Participants were excluded from the analyses where they had missing data. To address confounding due to genetic ancestry, we first stratified by self-reported race and analyzed each group separately, using the following linear model: baseline or percent change in serum urate = age + sex + systolic blood pressure (SBP) + BMI + SNP. Adopting a two-step approach, each of the aforementioned outcomes was first adjusted for all the covariates, except for SNP; the resulting residuals were then regressed on each of the 30 SNPs using an additive genetic model. The covariates were selected based on literature [17,18]. Subsequently, summary statistics from each racial group were meta-analyzed using fixed effects models applying inverse standard error weighting in METAL [19]. The heterogeneity statistics from METAL including Cochran’s Q-test are presented in the Supplementary Tables S1 and S2.

We chose the stratify-and-combine approach over a pooled analysis, because (1) we were not able to compute principal components, which are commonly included as model covariates to adjust for confounding due to genetic ancestry [20], and (2) no participant was excluded as racial outlier since all 57 participants self-identified as either Black or White. We collected race as a proxy for genetic ancestry, as race is associated with genetic ancestry, which is in turn related to genetic variants [21]. Statistical analyses were performed using R Statistical Software (v 3.6.2) [22]. Although we examined all nominally significant findings (*p* ≤ 0.05), we further corrected for multiple testing in the meta-analysis (*p* ≤ 0.00167), corresponding to a Bonferroni correction of 30 independent tests.

## 3. Results

A total of 57 participants (20 self-reported as Black) were enrolled and completed the fructose challenge. We were unable to measure serum urate in three participants, as two of them missed 30-min blood draws and one missed 180-min. At baseline, participants had a mean ± standard deviation age of 39.23 ± 6.81 years, BMI of 29.78 ± 7.50 kg/m^2^, and serum urate concentration of 5.52 ± 0.99 mg/dL (Table 1). Compared to men, women on average had significantly higher percent body fat (*p* < 0.0001), lower SBP (*p* = 0.01), and lower serum urate (*p* = 0.01). At baseline, participants who were White, men, or obese had higher serum urate levels than those who were Black (5.60 ± 1.01 vs. 5.37 ± 0.96), women (6.17 ± 1.14 vs. 5.24 ± 0.79), or had lower BMI (5.69 ± 1.08 vs. 5.42 ± 1.06 vs. 5.34 ± 0.80) (Table 2).

### 3.1. Associations between Baseline Serum Urate and SNPs

The effect allele frequencies (EAFs) and summary statistics on the associations between each of the 30 SNPs before the fructose challenge are shown in Table 3. The EAFs ranged from 0% to 79%, with wide differences between self-reported racial groups for some SNPs (e.g., EAF of rs2762353 was 5% in Black and 41% in White participants). In the baseline serum urate meta-analysis, SNP rs2941484 reached the prespecified multiple testing-corrected significance level. The effect allele C was negatively associated with baseline serum urate (EAF_total population_ = 37%, beta = −0.59, SE = 0.15, *p* = 1.37 × 10^−4^). The effects were consistent in the two racial groups for rs2941484 (beta_Black_ = −0.55, beta_White_ = −0.59, *p_heterogeneity_* = 0.94, Supplementary Table S1). 

### 3.2. Variations in Serum Urate within Three Hours of the Fructose Challenge

**Figure 2:**
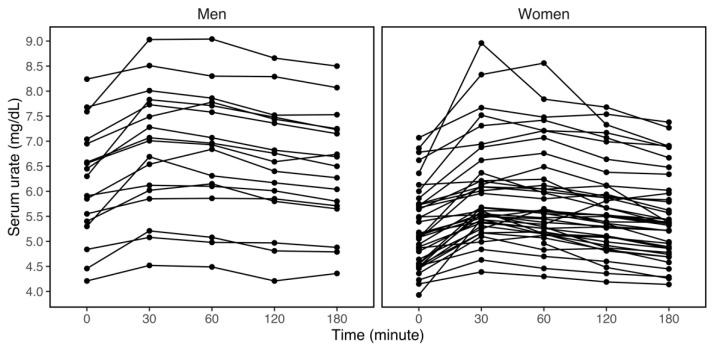
Individual trajectories of serum urate (mg/dL) within three hours after the fructose challenge, stratifying by sex (N_men_ = 17, N_women_ = 40). Each line represents one participant. For most participants, serum urate increased drastically in the first 30 min and then gradually declined towards baseline levels in the remaining 150 min. On average, men had higher serum urate concentrations throughout the three hours.

Although serum urate varied by individual within the three-hour observation period, most participants experienced a spike in their serum urate concentrations in the first 30 min, with an average increase of 12.63% (Figure 2). Stratifying further by race, sex, and BMI uncovered a similar pattern within each group—an increase and peak within the first 30 min, followed by a gradual decline towards baseline levels (Table 2). Analogous to what was observed at baseline, concentrations of serum urate remained higher in participants who were White, men, or those with obesity than those who were Black, women, or had lower BMI in these three hours (Table 2).

### 3.3. Associations between Percent Changes in Serum Urate and SNPs

In the meta-analysis, two SNPs were associated with one of the four percent changes in serum urate after multiple-testing correction (Table 4): rs7976059 (beta = −0.04, SE = 0.01, *p* = 1.07 × 10^−3^ during the first 30 min) and rs17050272 (beta = −0.02, SE = 0.01, *p* = 5.92 × 10^−5^ during 60–120 min). Additionally, six SNPs were nominally significant (Table 4); four of them were associated with percent change during 30–60 min, including rs2307394 (beta = 0.02, SE = 0.01, *p* = 0.01), rs1178977 (beta = −0.02, SE = 0.01, *p* = 0.02), rs10480300 (beta = −0.01, SE = 0.01, *p* = 0.05), rs1171614 (beta = −0.02, SE = 0.01, *p* = 0.01), rs1035942 (beta = 0.01, SE = 0.01, *p* = 0.03), while one of them, rs3775948, was associated with percent change during 60–120 min (beta −0.01, SE = 0.004, *p* = 1.73 × 10^−3^). The effect sizes were homogeneous across racial groups for four of the eight SNPs, except for rs7976059 (beta_Black_ = 0.002, beta_White_ = −0.06, *p_heterogeneity_* = 0.02), rs2307394 (beta_Black_ = 0.004, beta_White_ = 0.03, *p_heterogeneity_* = 0.03), rs3775948 (beta_Black_ = −0.02, beta_White_ = 0.01, *p_heterogeneity_* = 0.02), and rs17050272 (beta_Black_ = −0.03, beta_White_ = −0.01, *p_heterogeneity_* = 0.03) (Supplementary Table S2). The full results for all 30 SNPs are shown in Supplementary Table S2. 

## 4. Discussion

Our feasibility pilot study showed that when a SSB containing 80% fructose was consumed as the sole energy source, despite individual variations, serum urate escalated and then restored towards its baseline concentration. Three of the 30 SNPs reached multiple testing corrected significance, associated with baseline serum urate or serum percent changes, while six SNPs were nominally associated with serum urate percent changes. Serum urate concentrations are variable and under regulation of genetic and environmental factors [1]. Our results indicated that acute intervention studies such as ours are worth pursuing in the future, as these studies could offer insights into the short-term roles that genetic variants play in urate metabolism beyond traditional GWAS.

Our findings extended the results from previous controlled feeding studies, most of which investigated the effect of fructose-containing beverages on serum urate in relatively long-term settings [7,11,12], ranging from 2 weeks [11] to 6 months [12]. Thus, it was uncertain whether urate would rise when a single dose of fructose was consumed [23]. A systematic review and meta-analysis of 21 controlled feeding trials concluded that consuming hypercaloric fructose for longer than a week elevated urate, whereas isocaloric fructose did not [24]. Nonetheless, hypercaloric studies also suffered from confounding due to excess energy. Herein, by consuming the drink after an overnight fast, we isolated the impact of a SSB from the effect of extra calorie intake. We showed that a SSB offering 300 kcal would also elevate serum urate concentration. We replicated the response previously reported in Akhavan and Anderson [6] and Dalbeth et al. [10] who showed that a drink of 300 kcal/300 mL elicited an elevated urate response. It is also worth noting that the fructose concentration of 200 g/L used in current study is equivalent to 3 to 4 cans of soft drink products that have high-fructose corn syrup as an ingredient [25].

Two of the 30 SNPs included in this study were also examined in other studies using similar study designs. In a study of 74 participants from New Zealand, the T allele of rs2231142 on *ABCG2* (EAF 31%) was associated with a smaller increase in serum urate since 60 min after the fructose load until 180 min. In contrast, the T allele was only present in 8% of our White participants while absent in our Black participants, therefore the T allele was not significantly associated with any of the percent changes in serum urate. Another SNP, rs1183201, was also studied in the same group of New Zealand participants, with comparable allele frequencies of the A allele observed across all ancestral groups (EAF 62%) [9]. Dalbeth et al. described this A allele as ‘protective’ since A allele carriers had lower serum urate at all five time points after the fructose load [9]. In our study, the A allele was much more common in the White participants (EAF_White_ = 42% vs. EAF_Black_ = 8%) and we did not observe any significant protective effect conferred by the allele in the meta-analysis. The discrepancies between findings from these two studies and ours are likely due to differences in participant characteristics (e.g., EAF) and modeling approach where we used a two-step model to perform group-specific analyses and then meta-analyzed.

The nearest genes of the three SNPs that showed multiple testing corrected significance are *HNF4G*, *INHBB*, and *ACVR1B/ACVRL1*, respectively. SNP rs2941484, a 3 prime UTR variant located on *HNF4G*, has been reported repeatedly to be associated with urate in previous GWAS [14,26,27,28]. A member of orphan nuclear receptor family, HNF4G has been mainly implicated in lung and bladder cancer where it is believed to promote cell proliferation [29,30]. Studies have shown that HNF1 (HNF1a, HNF4a and HNF4G) set of transcription factors regulate serum urate through their effects on urate transporters [31,32]. GWAS have shown TT genotype of rs2941484 of *HNF4G* to be associated with elevated serum urate, hyperuricemia and/or gout [26,33,34]. This was replicated in our study where T allele was shown to be associated with elevated serum urate. Changes in serum urate in response to acute fructose load were also significantly associated with variants in *INHBB* and *ACVR1B/ACVRL1* genes. Inhibins are glycoproteins that are members of TGF-beta superfamily and are involved in activin/inhibin signaling. Genetic variants of *INHBB* have also been associated with preeclampsia, which has also been linked to altered serum urate levels [35,36]. Previous studies found the A allele of rs17050272 of *INHBB* to be associated with increased serum urate [14,26,32]. Our study found that the A allele was associated with a greater change in serum urate after an hour of SSB consumption, although baseline serum urate did not have a significant association. Another gene in the same TGF-beta superfamily, *ACRL1*, was also associated with percent changes in serum urate in our study. This gene encodes a type 1 cell-surface receptor whose sequence variants have been associated with hereditary hemorrhagic telangiectasia and Crohn’s disease. Variants in these genes were found to be associated with hyperuricemia and gout [14,37].

The six nominally significant SNPs have been reported to be associated with urate in GWAS [14,15] but none have been associated with percent change in urate. Our data with multiple time points painted a more dynamic picture of urate metabolism, implying the possible role of genetic variants or the underlying genes on serum urate regulation. Beyond the urate transporter loci like *ABCG2* and *SLC2A9*, little is known about other loci discovered in the past GWAS. Given that most of the urate-related SNPs residing in the non-coding regions of the genome [38], understanding their mechanisms is particularly challenging yet imperative, and using a study design analogous to ours could offer additional insights.

Our results should be interpreted considering the following strengths and limitations. First, to the best of our knowledge, this is the first intervention study that compared the acute effect of a SSB on serum urate between Black and White Americans. Previous studies with similar study designs compared between Māori or Pacific Ancestry, Eastern Polynesian, Western Polynesian, and European ancestries [8,9,10]. Secondly, all these studies investigated a single SNP while we expanded to 30 urate-related SNPs. We also recognize that there are limitations. Since this was a pilot and feasibility study, our small sample size and the relatively short duration limited our ability to detect possible effects of SSB on serum urate. Furthermore, incorporating 15 g of glucose per drink to alleviate gastrointestinal discomfort may have failed to evaluate the effects of fructose in isolation. Finally, we did not collect fecal or urine samples since the primary outcome of this study was to understand the effects of an acute fructose challenge on serum urate response.

## 5. Conclusions

In conclusion, our results showed that our study design was feasible, which could offer additional insights beyond studies using cross-sectional urate measurements. Additionally, we showed that serum urate rose and fell within three hours of SSB consumption and genetic variants could play a role in modulating the concentration of serum urate in response to an acute fructose load.

## Figures and Tables

**Table 1:** Study participants characteristics at baseline.

	Total	Men	Women	*p*
N	57	17	40	
Age, years	39.23 (6.81)	38.71 (8.37)	39.45 (6.14)	0.74
Weight, kg	84.56 (21.66)	90.77 (16.51)	81.92 (23.19)	0.11
BMI, kg/m^2^	29.78 (7.50)	28.97 (5.05)	30.12 (8.36)	0.53
PBF, %	34.65 (10.59)	25.51 (8.22)	38.53 (9.02)	<0.0001
WC, cm	97.02 (17.75)	100.03 (13.27)	95.74 (19.35)	0.34
WHtR	0.58 (0.11)	0.57 (0.08)	0.58 (0.12)	0.59
SBP, mmHg	115.91 (14.12)	123.06 (11.01)	112.88 (14.31)	0.01
DBP, mmHg	74.57 (11.51)	75.35 (12.93)	74.24 (11.01)	0.76
Serum urate, mg/dL	5.52 (0.99)	6.17 (1.14)	5.24 (0.79)	0.01

Values are presented as mean (SD). BMI, body mass index; PBF, percent body fat; WC, weight circumference; WHtR, waist-to-height ratio; SBP, systolic blood pressure; DBP, diastolic blood pressure.

**Table 2:** Serum urate (mg/dL) at five time points, by race, sex, and BMI group.

Groups	Subgroups	N	Time (minute)				
			0	30	60	120	180
Race	Black	20	5.37 (0.96)	5.80 (1.06)	5.69 (1.05)	5.54 (1.04)	5.42 (1.02)
	White	37	5.60 (1.01)	6.44 (1.13)	6.34 (1.11)	6.12 (1.03)	5.93 (1.02)
Sex	Men	17	6.17 (1.14)	6.82 (1.25)	6.77 (1.24)	6.54 (1.21)	6.42 (1.15)
	Women	40	5.24 (0.79)	5.95 (0.99)	5.84 (0.96)	5.65 (0.88)	5.47 (0.85)
BMI	Normal	18	5.34 (0.80)	6.21 (1.05)	6.12 (1.02)	5.84 (0.86)	5.65 (0.81)
	Overweight	14	5.42 (1.06)	6.16 (1.30)	6.08 (1.29)	5.82 (1.24)	5.69 (1.21)
	Obese	25	5.69 (1.08)	6.25 (1.16)	6.12 (1.15)	6.02 (1.12)	5.88 (1.10)

Values are shown as mean (SD). BMI, body mass index.

**Table 3:** Associations between SNPs and baseline serum urate.

Nearest Gene	SNP	Allele		Black (N = 20)					White (N = 37)					Meta-Analysis			
		Effect	Other	EAF	Beta	SE	*p*		EAF	Beta	SE	*p*		EAF	Beta	SE	*p*
*ABCG2*	rs2231142	T	G	0.00	n.a	n.a	n.a		8.11	−0.01	0.36	0.98		5.36	−0.01	0.36	0.98
*SLC2A9*	rs16890979	T	C	52.50	0.16	0.32	0.62		17.57	−0.34	0.24	0.17		29.82	−0.15	0.19	0.43
*SLC17A1*	rs1183201	A	T	7.50	0.31	0.59	0.60		41.89	0.11	0.18	0.56		29.82	0.13	0.18	0.47
*SLC2A9*	rs737267	T	G	52.63	0.30	0.32	0.36		20.27	−0.40	0.24	0.10		31.25	−0.15	0.19	0.42
*SLC2A9*	rs6449213	C	T	26.32	−0.20	0.37	0.61		14.86	−0.22	0.26	0.40		18.75	−0.21	0.21	0.32
*SLC2A9*	rs3775948	C	G	39.47	−0.32	0.31	0.33		18.92	−0.32	0.22	0.15		25.89	−0.32	0.18	0.08
*TRIM46*	rs11264341	T	C	23.68	0.09	0.45	0.84		48.65	−0.08	0.19	0.66		40.18	−0.06	0.17	0.74
*INHBB*	rs17050272	A	G	10.53	−0.52	0.41	0.22		51.35	−0.10	0.21	0.63		37.50	−0.19	0.19	0.32
*ORC4*	rs2307394	G	A	23.68	0.20	0.45	0.66		29.73	0.06	0.22	0.78		27.68	0.09	0.20	0.65
*LRRC16A*	rs9358856	A	G	10.53	−0.27	0.55	0.63		14.86	0.15	0.29	0.61		13.39	0.06	0.26	0.82
*SLC17A3*	rs2762353	T	C	5.26	0.53	0.72	0.48		40.54	0.13	0.18	0.49		28.57	0.15	0.18	0.40
*SLC17A1*	rs1165151	A	C	7.89	0.33	0.61	0.60		41.89	0.11	0.18	0.56		30.36	0.13	0.18	0.47
*SLC22A7*	rs4149178	G	A	31.58	−0.12	0.30	0.69		14.86	−0.02	0.26	0.93		20.54	−0.07	0.20	0.74
*BAZ1B*	rs1178977	G	A	21.05	−0.38	0.37	0.31		6.76	−0.29	0.39	0.46		11.61	−0.34	0.27	0.21
*PRKAG2*	rs10480300	T	C	36.84	−0.09	0.31	0.77		17.57	0.00	0.28	1.00		24.11	−0.04	0.21	0.85
*MBOAT4*	rs7813902	T	C	21.05	−0.29	0.37	0.46		2.70	0.32	0.59	0.59		8.93	−0.11	0.32	0.73
*HNF4G*	rs2941484	C	T	15.79	−0.55	0.47	0.25		47.30	−0.59	0.16	9.10 × 10^−4^		36.61	−0.59	0.15	1.37 × 10^−4^ **
*A1CF*	rs10821905	A	G	31.58	0.08	0.34	0.82		16.22	0.24	0.21	0.26		21.43	0.19	0.18	0.28
*SLC16A9*	rs12356193	G	A	7.89	−0.10	0.62	0.88		20.27	−0.10	0.22	0.67		16.07	−0.10	0.21	0.65
*SLC16A9*	rs1171614	A	G	23.68	−0.27	0.45	0.55		25.68	−0.08	0.22	0.72		25.00	−0.12	0.20	0.55
*NRXN2*	rs478607	G	A	55.26	0.50	0.29	0.10		20.27	−0.18	0.22	0.43		32.14	0.08	0.18	0.66
*RERG*	rs11056399	T	C	47.37	−0.17	0.27	0.53		36.49	0.11	0.21	0.59		40.18	0.01	0.16	0.97
*ACVR1B/ACVRL1*	rs7976059	T	G	18.42	−0.19	0.38	0.62		39.19	−0.08	0.19	0.68		32.14	−0.10	0.17	0.55
*INHBC*	rs3741414	A	G	13.16	−0.10	0.51	0.85		22.97	−0.13	0.19	0.51		19.64	−0.12	0.18	0.49
*B3GNT4*	rs7953704	A	G	36.84	−0.03	0.51	0.95		48.65	0.14	0.19	0.47		44.64	0.12	0.18	0.51
*IGF1R*	rs6598541	A	G	57.89	−0.09	0.34	0.78		24.32	0.02	0.21	0.91		35.71	−0.01	0.18	0.96
*UMOD*	rs4293393	C	T	15.79	0.29	0.39	0.46		25.68	0.01	0.19	0.97		22.32	0.06	0.17	0.72
*HLF*	rs7224610	C	A	0.00	n.a	n.a	n.a		32.43	−0.07	0.20	0.74		21.43	−0.07	0.20	0.74
*QRICH2*	rs164009	G	A	78.95	−0.46	0.44	0.31		28.38	−0.18	0.21	0.38		45.54	−0.23	0.19	0.21
*INSR*	rs1035942	T	C	34.21	0.31	0.39	0.44		32.43	0.10	0.17	0.55		33.04	0.14	0.16	0.38

SNP, single nucleotide polymorphism; EAF, effect allele frequency (%); SE, standard error; *p*, *p*-value; n.a., not available. ** Significant after multiple-testing correction (*p* < 0.00167) in the meta-analysis. Model (baseline serum urate = SNP + sex + age + systolic blood pressure + body mass index) was fitted using a two-step approach, followed by meta-analysis.

**Table 4:** Associations between SNPs and percent changes in serum urate for SNPs that reached statistical significance.

Nearest Gene	SNP	Allele		Time Period (minute)	Black (N = 20)					White (N = 37)					Meta-Analysis			
		Effect	Other		EAF	Beta	SE	*p*		EAF	Beta	SE	*p*		EAF	Beta	SE	*p*
*SLC2A9*	rs3775948	C	G	0–30	39.47	−0.01	0.02	0.55		18.92	0.01	0.02	0.64		25.89	0.00	0.02	0.89
*INHBB*	rs17050272	A	G		10.53	−0.01	0.04	0.85		51.35	0.00	0.02	0.88		37.50	0.00	0.02	0.83
*ORC4*	rs2307394	G	A		23.68	0.01	0.03	0.83		29.73	−0.01	0.02	0.58		27.68	−0.01	0.02	0.77
*BAZ1B*	rs1178977	G	A		21.05	0.03	0.02	0.12		6.76	0.00	0.04	0.93		11.61	0.03	0.02	0.16
*PRKAG2*	rs10480300	T	C		36.84	0.01	0.02	0.74		17.57	−0.03	0.03	0.30		24.11	0.00	0.02	0.76
*SLC16A9*	rs1171614	A	G		23.68	0.06	0.02	0.02		25.68	−0.01	0.02	0.52		25.00	0.02	0.02	0.16
*ACVR1B/ACVRL1*	rs7976059	T	G		18.42	0.00	0.02	0.93		39.19	−0.06	0.02	3.29 × 10^−4^		32.14	−0.04	0.01	1.07 × 10^−3^ **
*INSR*	rs1035942	T	C		34.21	−0.05	0.02	0.03		32.43	−0.01	0.02	0.60		33.04	−0.03	0.01	0.05
*SLC2A9*	rs3775948	C	G	30–60	39.47	0.00	0.01	0.76		18.92	−0.01	0.01	0.42		25.89	0.00	0.01	0.84
*INHBB*	rs17050272	A	G		10.53	−0.01	0.02	0.70		51.35	0.00	0.01	0.93		37.50	0.00	0.01	0.89
*ORC4*	rs2307394	G	A		23.68	0.00	0.01	0.68		29.73	0.03	0.01	1.72 × 10^−3^		27.68	0.02	0.01	0.01 *
*BAZ1B*	rs1178977	G	A		21.05	−0.01	0.01	0.06		6.76	−0.02	0.02	0.23		11.61	−0.02	0.01	0.02 *
*PRKAG2*	rs10480300	T	C		36.84	−0.01	0.01	0.09		17.57	−0.01	0.01	0.38		24.11	−0.01	0.01	0.05 *
*SLC16A9*	rs1171614	A	G		23.68	−0.02	0.01	0.02		25.68	−0.01	0.01	0.19		25.00	−0.02	0.01	0.01 *
*ACVR1B/ACVRL1*	rs7976059	T	G		18.42	0.00	0.01	0.55		39.19	0.01	0.01	0.19		32.14	0.01	0.01	0.18
*INSR*	rs1035942	T	C		34.21	0.01	0.01	0.12		32.43	0.01	0.01	0.16		33.04	0.01	0.01	0.03 *
*SLC2A9*	rs3775948	C	G	60–120	39.47	−0.02	0.01	0.00		18.92	0.01	0.01	0.57		25.89	−0.01	0.00	1.73 × 10^−3^ *
*INHBB*	rs17050272	A	G		10.53	−0.03	0.01	0.00		51.35	−0.01	0.01	0.51		37.50	−0.02	0.01	5.92 × 10^−5^ **
*ORC4*	rs2307394	G	A		23.68	−0.01	0.01	0.49		29.73	−0.01	0.01	0.44		27.68	−0.01	0.01	0.29
*BAZ1B*	rs1178977	G	A		21.05	0.00	0.01	0.73		6.76	0.02	0.02	0.23		11.61	0.00	0.01	0.80
*PRKAG2*	rs10480300	T	C		36.84	0.00	0.01	0.64		17.57	0.02	0.01	0.11		24.11	0.00	0.01	0.64
*SLC16A9*	rs1171614	A	G		23.68	0.01	0.01	0.35		25.68	0.00	0.01	0.66		25.00	0.01	0.01	0.32
*ACVR1B/ACVRL1*	rs7976059	T	G		18.42	0.00	0.01	0.73		39.19	0.00	0.01	0.79		32.14	0.00	0.01	0.66
*INSR*	rs1035942	T	C		34.21	0.01	0.01	0.20		32.43	0.00	0.01	0.85		33.04	0.00	0.01	0.48
*SLC2A9*	rs3775948	C	G	120–180	39.47	0.00	0.00	0.97		18.92	0.00	0.01	0.86		25.89	0.00	0.00	0.90
*INHBB*	rs17050272	A	G		10.53	0.00	0.01	0.50		51.35	−0.01	0.01	0.34		37.50	0.00	0.00	0.93
*ORC4*	rs2307394	G	A		23.68	0.00	0.01	0.96		29.73	−0.01	0.01	0.23		27.68	0.00	0.00	0.46
*BAZ1B*	rs1178977	G	A		21.05	0.01	0.00	0.16		6.76	0.00	0.01	0.84		11.61	0.01	0.00	0.15
*PRKAG2*	rs10480300	T	C		36.84	0.00	0.00	0.31		17.57	0.02	0.01	0.05		24.11	0.00	0.00	0.95
*SLC16A9*	rs1171614	A	G		23.68	0.00	0.01	0.44		25.68	0.00	0.01	0.96		25.00	0.00	0.00	0.52
*ACVR1B/ACVRL1*	rs7976059	T	G		18.42	0.00	0.00	0.96		39.19	0.01	0.01	0.07		32.14	0.00	0.00	0.21
*INSR*	rs1035942	T	C		34.21	0.00	0.00	0.81		32.43	0.00	0.01	0.34		33.04	0.00	0.00	0.62

SNP, single nucleotide polymorphism; EAF, effect allele frequency (%); SE, standard error; *p*, *p*-value; n.a., not available. ** Significant after multiple-testing correction (*p* < 0.00167) in the meta-analysis; * Nominal significance (*p* < 0.05) in the meta-analysis. Model (percent change in serum urate = SNP + sex + age + systolic blood pressure + body mass index) was fitted using a two-step approach, followed by meta-analysis.

## Data Availability

Data described in the manuscript, code book and analytic code will be made available upon request pending approval with corresponding author.

## Supplementary Materials

The following supporting information can be downloaded at: https://www.mdpi.com/article/10.3390/nu14194030/s1, Table S1: Associations between SNPs and baseline serum urate, including heterogeneity statistics from METAL; Table S2: Associations between SNPs and percent changes in serum urate, including heterogeneity statistics from METAL.
