# Supplementary material for: Novel Insights into the Effects of Genetic Variants on Serum Urate Response to an Acute Fructose Challenge: A Pilot Study
Source: Nutrients. 2022 Sep 28;14(19):4030. doi: 10.3390/nu14194030 (PMC9570712; doi:10.3390/nu14194030)
Supplement: Supplementary file 1 [file nutrients-14-04030-s001.zip › Supplementary Table S1_09242022.pdf]

Supplementary Table S1. Associations between SNPs and baseline serum urate, including heterogeneity statistics from METAL

Nearest gene	SNP	Allele		Black (N = 20)				White (N = 37)				Meta-analysis								
		E	O	EAF	Beta	SE	*P*	EAF	Beta	SE	*P*	EAF	Beta	SE	*P*	D	I^2	ChiSq	DF	Het*P*
ABCG2	rs2231142	T	G	0.00	n.a	n.a	n.a	8.11	-0.01	0.36	0.98	5.36	-0.01	0.36	0.98	?-	0	0.00	0	1.00
SLC2A9	rs16890979	T	C	52.50	0.16	0.32	0.62	17.57	-0.34	0.24	0.17	29.82	-0.15	0.19	0.43	+-	35.4	1.55	1	0.21
SLC17A1	rs1183201	A	T	7.50	0.31	0.59	0.60	41.89	0.11	0.18	0.56	29.82	0.13	0.18	0.47	++	0	0.11	1	0.74
SLC2A9	rs737267	T	G	52.63	0.30	0.32	0.36	20.27	-0.40	0.24	0.10	31.25	-0.15	0.19	0.42	+-	67.5	3.07	1	0.08
SLC2A9	rs6449213	C	T	26.32	-0.20	0.37	0.61	14.86	-0.22	0.26	0.40	18.75	-0.21	0.21	0.32	--	0	0.00	1	0.96
SLC2A9	rs3775948	C	G	39.47	-0.32	0.31	0.33	18.92	-0.32	0.22	0.15	25.89	-0.32	0.18	0.08	--	0	0.00	1	0.99
TRIM46	rs11264341	T	C	23.68	0.09	0.45	0.84	48.65	-0.08	0.19	0.66	40.18	-0.06	0.17	0.74	+-	0	0.12	1	0.73
INHBB	rs17050272	A	G	10.53	-0.52	0.41	0.22	51.35	-0.10	0.21	0.63	37.50	-0.19	0.19	0.32	--	0	0.82	1	0.36
ORC4	rs2307394	G	A	23.68	0.20	0.45	0.66	29.73	0.06	0.22	0.78	27.68	0.09	0.20	0.65	++	0	0.08	1	0.78
LRRC16A	rs9358856	A	G	10.53	-0.27	0.55	0.63	14.86	0.15	0.29	0.61	13.39	0.06	0.26	0.82	-+	0	0.45	1	0.50
SLC17A3	rs2762353	T	C	5.26	0.53	0.72	0.48	40.54	0.13	0.18	0.49	28.57	0.15	0.18	0.40	++	0	0.29	1	0.59
SLC17A1	rs1165151	A	C	7.89	0.33	0.61	0.60	41.89	0.11	0.18	0.56	30.36	0.13	0.18	0.47	++	0	0.11	1	0.74
SLC22A7	rs4149178	G	A	31.58	-0.12	0.30	0.69	14.86	-0.02	0.26	0.93	20.54	-0.07	0.20	0.74	--	0	0.07	1	0.80
BAZ1B	rs1178977	G	A	21.05	-0.38	0.37	0.31	6.76	-0.29	0.39	0.46	11.61	-0.34	0.27	0.21	--	0	0.03	1	0.85
PRKAG2	rs10480300	T	C	36.84	-0.09	0.31	0.77	17.57	0.00	0.28	1.00	24.11	-0.04	0.21	0.85	-+	0	0.05	1	0.82
MBOAT4	rs7813902	T	C	21.05	-0.29	0.37	0.46	2.70	0.32	0.59	0.59	8.93	-0.11	0.32	0.73	-+	0	0.75	1	0.39
HNF4G	rs2941484	C	T	15.79	-0.55	0.47	0.25	47.30	-0.59	0.16	9.10E -04	36.61	-0.59	0.15	1.37E -04**	--	0	0.01	1	0.94
A1CF	rs10821905	A	G	31.58	0.08	0.34	0.82	16.22	0.24	0.21	0.26	21.43	0.19	0.18	0.28	++	0	0.17	1	0.68
SLC16A9	rs12356193	G	A	7.89	-0.10	0.62	0.88	20.27	-0.10	0.22	0.67	16.07	-0.10	0.21	0.65	--	0	0.00	1	1.00
SLC16A9	rs1171614	A	G	23.68	-0.27	0.45	0.55	25.68	-0.08	0.22	0.72	25.00	-0.12	0.20	0.55	--	0	0.15	1	0.70
NRXN2	rs478607	G	A	55.26	0.50	0.29	0.10	20.27	-0.18	0.22	0.43	32.14	0.08	0.18	0.66	+-	71.4	3.49	1	0.06
RERG	rs11056399	T	C	47.37	-0.17	0.27	0.53	36.49	0.11	0.21	0.59	40.18	0.01	0.16	0.97	-+	0	0.71	1	0.40
ACVR1B/ ACVRL1	rs7976059	T	G	18.42	-0.19	0.38	0.62	39.19	-0.08	0.19	0.68	32.14	-0.10	0.17	0.55	--	0	0.07	1	0.79
INHBC	rs3741414	A	G	13.16	-0.10	0.51	0.85	22.97	-0.13	0.19	0.51	19.64	-0.12	0.18	0.49	--	0	0.00	1	0.96
B3GNT4	rs7953704	A	G	36.84	-0.03	0.51	0.95	48.65	0.14	0.19	0.47	44.64	0.12	0.18	0.51	-+	0	0.10	1	0.75
IGF1R	rs6598541	A	G	57.89	-0.09	0.34	0.78	24.32	0.02	0.21	0.91	35.71	-0.01	0.18	0.96	-+	0	0.09	1	0.76
UMOD	rs4293393	C	T	15.79	0.29	0.39	0.46	25.68	0.01	0.19	0.97	22.32	0.06	0.17	0.72	++	0	0.43	1	0.51
HLF	rs7224610	C	A	0.00	n.a	n.a	n.a	32.43	-0.07	0.20	0.74	21.43	-0.07	0.20	0.74	?-	0	0.00	0	1.00
QRICH2	rs164009	G	A	78.95	-0.46	0.44	0.31	28.38	-0.18	0.21	0.38	45.54	-0.23	0.19	0.21	--	0	0.32	1	0.57
INSR	rs1035942	T	C	34.21	0.31	0.39	0.44	32.43	0.10	0.17	0.55	33.04	0.14	0.16	0.38	++	0	0.24	1	0.63

SNP, single nucleotide polymorphism; E, effect allele; O, other allele; EAF, effect allele frequency (%); SE, standard error; P, p-value; D, direction of effect; ChiSq, heterogeneity test statistic; DF, degrees of freedom for heterogeneity test; HetP, heterogeneity p-value; n.a., not available.

** Significant after multiple-testing correction ($p < 0.00167$) in the meta-analysis.

Model (baseline serum urate = age + sex + systolic blood pressure + body mass index + SNP) was fitted using a 2-step approach, followed by meta-analysis.
