# Supplementary material for: Novel Insights into the Effects of Genetic Variants on Serum Urate Response to an Acute Fructose Challenge: A Pilot Study
Source: Nutrients. 2022 Sep 28;14(19):4030. doi: 10.3390/nu14194030 (PMC9570712; doi:10.3390/nu14194030)
Supplement: Supplementary file 1 [file nutrients-14-04030-s001.zip › Supplementary Table S2_09242022.pdf]

Supplementary Table S2. Associations between SNPs and percent changes in serum urate, including heterogeneity statistics from METAL

				0 – 30 minutes																
Nearest gene	SNP	Allele		Black (N = 20)				White (N = 37)				Meta-analysis								
		E	O	EAF	Beta	SE	*P*	EAF	Beta	SE	*P*	EAF	Beta	SE	*P*	D	I^2	ChiSq	DF	Het*P*
ABCG2	rs2231142	T	G	0.00	n.a	n.a	n.a	8.11	-0.06	0.03	0.09	5.36	-0.06	0.03	0.09	?-	0	0.00	0	1.00
SLC2A9	rs16890979	T	C	52.50	0.00	0.02	0.84	17.57	0.02	0.02	0.40	29.82	0.01	0.02	0.72	-+	0	0.65	1	0.42
SLC17A1	rs1183201	A	T	7.50	-0.01	0.04	0.83	41.89	0.02	0.02	0.20	29.82	0.02	0.02	0.27	-+	0	0.53	1	0.47
SLC2A9	rs737267	T	G	52.63	0.00	0.02	0.82	20.27	0.03	0.02	0.26	31.25	0.01	0.02	0.57	-+	5.7	1.06	1	0.30
SLC2A9	rs6449213	C	T	26.32	-0.01	0.02	0.81	14.86	0.01	0.03	0.63	18.75	0.00	0.02	0.89	-+	0	0.28	1	0.60
SLC2A9	rs3775948	C	G	39.47	-0.01	0.02	0.55	18.92	0.01	0.02	0.64	25.89	0.00	0.02	0.89	-+	0	0.59	1	0.44
TRIM46	rs11264341	T	C	23.68	-0.03	0.03	0.34	48.65	-0.01	0.02	0.60	40.18	-0.02	0.02	0.32	--	0	0.24	1	0.62
INHBB	rs17050272	A	G	10.53	-0.01	0.04	0.85	51.35	0.00	0.02	0.88	37.50	0.00	0.02	0.83	--	0	0.01	1	0.92
ORC4	rs2307394	G	A	23.68	0.01	0.03	0.83	29.73	-0.01	0.02	0.58	27.68	-0.01	0.02	0.77	+-	0	0.28	1	0.59
LRRC16A	rs9358856	A	G	10.53	-0.05	0.03	0.09	14.86	-0.01	0.03	0.71	13.39	-0.03	0.02	0.12	--	2.5	1.03	1	0.31
SLC17A3	rs2762353	T	C	5.26	-0.02	0.06	0.68	40.54	0.02	0.02	0.32	28.57	0.01	0.02	0.41	-+	0	0.49	1	0.48
SLC17A1	rs1165151	A	C	7.89	-0.01	0.04	0.76	41.89	0.02	0.02	0.20	30.36	0.02	0.02	0.29	-+	0	0.65	1	0.42
SLC22A7	rs4149178	G	A	31.58	0.02	0.02	0.40	14.86	-0.02	0.03	0.42	20.54	0.00	0.01	0.80	+-	25.5	1.34	1	0.25
BAZ1B	rs1178977	G	A	21.05	0.03	0.02	0.12	6.76	0.00	0.04	0.93	11.61	0.03	0.02	0.16	+-	0	0.72	1	0.40
PRKAG2	rs10480300	T	C	36.84	0.01	0.02	0.74	17.57	-0.03	0.03	0.30	24.11	0.00	0.02	0.76	+-	11.8	1.13	1	0.29
MBOAT4	rs7813902	T	C	21.05	0.01	0.02	0.62	2.70	0.04	0.06	0.53	8.93	0.01	0.02	0.49	++	0	0.16	1	0.69
HNF4G	rs2941484	C	T	15.79	0.02	0.03	0.59	47.30	0.02	0.02	0.24	36.61	0.02	0.02	0.19	++	0	0.04	1	0.84
A1CF	rs10821905	A	G	31.58	0.00	0.02	0.93	16.22	0.02	0.02	0.31	21.43	0.01	0.01	0.44	++	0	0.48	1	0.49
SLC16A9	rs12356193	G	A	7.89	0.01	0.04	0.77	20.27	-0.01	0.02	0.71	16.07	0.00	0.02	0.87	+-	0	0.21	1	0.65
SLC16A9	rs1171614	A	G	23.68	0.06	0.02	0.02	25.68	-0.01	0.02	0.52	25.00	0.02	0.02	0.16	+-	81.5	5.40	1	0.02
NRXN2	rs478607	G	A	55.26	0.01	0.02	0.50	20.27	0.04	0.02	0.11	32.14	0.02	0.01	0.10	++	0	0.61	1	0.44
RERG	rs11056399	T	C	47.37	0.02	0.02	0.25	36.49	0.02	0.02	0.39	40.18	0.02	0.01	0.14	++	0	0.00	1	0.96
ACVR1B/ ACVRL1	rs7976059	T	G	18.42	0.00	0.02	0.93	39.19	-0.06	0.02	3.29 E-04	32.14	-0.04	0.01	1.07E -03**	+-	81	5.26	1	0.02
INHBC	rs3741414	A	G	13.16	0.00	0.03	0.95	22.97	0.01	0.02	0.51	19.64	0.01	0.02	0.60	-+	0	0.17	1	0.68
B3GNT4	rs7953704	A	G	36.84	0.00	0.03	0.97	48.65	-0.01	0.02	0.51	44.64	-0.01	0.02	0.56	--	0	0.11	1	0.74
IGF1R	rs6598541	A	G	57.89	0.00	0.02	0.93	24.32	-0.02	0.02	0.34	35.71	-0.01	0.01	0.46	--	0	0.42	1	0.52
UMOD	rs4293393	C	T	15.79	0.00	0.02	0.86	25.68	0.01	0.02	0.61	22.32	0.00	0.02	0.78	-+	0	0.22	1	0.64
HLF	rs7224610	C	A	0.00	n.a	n.a	n.a	32.43	0.00	0.02	0.98	21.43	0.00	0.02	0.98	?-	0	0.00	0	1.00
QRICH2	rs164009	G	A	78.95	-0.01	0.03	0.64	28.38	0.03	0.02	0.12	45.54	0.02	0.02	0.31	-+	44.7	1.81	1	0.18
INSR	rs1035942	T	C	34.21	-0.05	0.02	0.03	32.43	-0.01	0.02	0.60	33.04	-0.03	0.01	0.05	--	56.2	2.29	1	0.13

SNP, single nucleotide polymorphism; E, effect allele; O, other allele; EAF, effect allele frequency (%); SE, standard error; P, p-value; D, direction of effect; ChiSq, heterogeneity test statistic, DF, degrees of freedom for heterogeneity test; HetP, heterogeneity p-value; n.a., not available.

**Significant after multiple-testing correction ($p < 0.00167$) in the meta-analysis; *Nominal significance ($p < 0.05$) in the meta-analysis

Model (percent change in serum urate = age + sex + systolic blood pressure + body mass index + SNP) was fitted using a 2-step approach, followed by meta-analysis.

Supplementary Table 2. Associations between SNPs and percent changes in serum urate, including heterogeneity statistics from METAL (cont.)

				30 – 60 minutes																
Nearest gene	SNP	Allele		Black (N = 20)				White (N = 37)				Meta-analysis								
		E	O	EAF	Beta	SE	*P*	EAF	Beta	SE	*P*	EAF	Beta	SE	*P*	D	I^2	ChiSq	DF	Het*P*
ABCG2	rs2231142	T	G	0.00	n.a	n.a	n.a	8.11	0.00	0.02	0.90	5.36	0.00	0.02	0.90	?-	0	0.00	0	1.00
SLC2A9	rs16890979	T	C	52.50	0.01	0.01	0.22	17.57	0.00	0.01	0.92	29.82	0.01	0.01	0.28	+-	0	0.44	1	0.51
SLC17A1	rs1183201	A	T	7.50	0.00	0.01	0.98	41.89	0.00	0.01	0.86	29.82	0.00	0.01	0.89	+-	0	0.01	1	0.91
SLC2A9	rs737267	T	G	52.63	0.01	0.01	0.35	20.27	-0.01	0.01	0.39	31.25	0.00	0.01	0.69	+-	35.1	1.54	1	0.21
SLC2A9	rs6449213	C	T	26.32	0.01	0.01	0.41	14.86	-0.01	0.01	0.44	18.75	0.00	0.01	0.76	+-	19.2	1.24	1	0.27
SLC2A9	rs3775948	C	G	39.47	0.00	0.01	0.76	18.92	-0.01	0.01	0.42	25.89	0.00	0.01	0.84	+-	0	0.73	1	0.39
TRIM46	rs11264341	T	C	23.68	0.00	0.01	0.96	48.65	-0.01	0.01	0.32	40.18	0.00	0.01	0.49	+-	0	0.52	1	0.47
INHBB	rs17050272	A	G	10.53	-0.01	0.02	0.70	51.35	0.00	0.01	0.93	37.50	0.00	0.01	0.89	-+	0	0.14	1	0.71
ORC4	rs2307394	G	A	23.68	0.00	0.01	0.68	29.73	0.03	0.01	1.72 E-03	27.68	0.02	0.01	0.01*	++	77.7	4.49	1	0.03
LRRC16A	rs9358856	A	G	10.53	0.00	0.01	0.70	14.86	0.02	0.01	0.15	13.39	0.01	0.01	0.53	-+	47.6	1.91	1	0.17
SLC17A3	rs2762353	T	C	5.26	0.00	0.02	0.94	40.54	0.00	0.01	0.92	28.57	0.00	0.01	0.90	++	0	0.00	1	0.97
SLC17A1	rs1165151	A	C	7.89	0.00	0.02	0.98	41.89	0.00	0.01	0.86	30.36	0.00	0.01	0.87	--	0	0.01	1	0.94
SLC22A7	rs4149178	G	A	31.58	0.00	0.01	0.98	14.86	0.00	0.01	0.91	20.54	0.00	0.01	0.97	-+	0	0.01	1	0.91
BAZ1B	rs1178977	G	A	21.05	-0.01	0.01	0.06	6.76	-0.02	0.02	0.23	11.61	-0.02	0.01	0.02	--	0	0.18	1	0.67
PRKAG2	rs10480300	T	C	36.84	-0.01	0.01	0.09	17.57	-0.01	0.01	0.38	24.11	-0.01	0.01	0.05*	--	0	0.01	1	0.92
MBOAT4	rs7813902	T	C	21.05	0.00	0.01	0.66	2.70	0.01	0.03	0.78	8.93	0.00	0.01	0.61	++	0	0.02	1	0.88
HNF4G	rs2941484	C	T	15.79	0.00	0.01	0.91	47.30	-0.01	0.01	0.36	36.61	0.00	0.01	0.55	+-	0	0.51	1	0.47
A1CF	rs10821905	A	G	31.58	0.00	0.01	0.52	16.22	-0.01	0.01	0.41	21.43	0.00	0.01	0.93	+-	10.4	1.12	1	0.29
SLC16A9	rs12356193	G	A	7.89	-0.01	0.01	0.31	20.27	-0.01	0.01	0.65	16.07	-0.01	0.01	0.30	--	0	0.23	1	0.63
SLC16A9	rs1171614	A	G	23.68	-0.02	0.01	0.02	25.68	-0.01	0.01	0.19	25.00	-0.02	0.01	0.01*	--	0	0.19	1	0.66
NRXN2	rs478607	G	A	55.26	0.00	0.01	0.55	20.27	-0.01	0.01	0.37	32.14	-0.01	0.01	0.32	--	0	0.20	1	0.66
RERG	rs11056399	T	C	47.37	0.00	0.01	0.64	36.49	-0.01	0.01	0.41	40.18	0.00	0.01	0.40	--	0	0.23	1	0.64
ACVR1B/ ACVRL1	rs7976059	T	G	18.42	0.00	0.01	0.55	39.19	0.01	0.01	0.19	32.14	0.01	0.01	0.18	++	0	0.35	1	0.55
INHBC	rs3741414	A	G	13.16	0.01	0.01	0.21	22.97	0.00	0.01	0.70	19.64	0.01	0.01	0.24	++	0	0.48	1	0.49
B3GNT4	rs7953704	A	G	36.84	0.00	0.01	0.66	48.65	-0.01	0.01	0.35	44.64	-0.01	0.01	0.32	--	0	0.10	1	0.75
IGF1R	rs6598541	A	G	57.89	0.00	0.01	0.54	24.32	-0.01	0.01	0.28	35.71	0.00	0.01	0.92	+-	36.5	1.58	1	0.21
UMOD	rs4293393	C	T	15.79	0.00	0.01	0.83	25.68	0.00	0.01	0.63	22.32	0.00	0.01	0.63	--	0	0.05	1	0.82
HLF	rs7224610	C	A	0.00	n.a	n.a	n.a	32.43	-0.01	0.01	0.28	21.43	-0.01	0.01	0.27	?-	0	0.00	0	1.00
QRICH2	rs164009	G	A	78.95	0.01	0.01	0.11	28.38	0.00	0.01	0.78	45.54	0.01	0.01	0.27	+-	42.8	1.75	1	0.19
INSR	rs1035942	T	C	34.21	0.01	0.01	0.12	32.43	0.01	0.01	0.16	33.04	0.01	0.01	0.03*	++	0	0.01	1	0.93

SNP, single nucleotide polymorphism; E, effect allele; O, other allele; EAF, effect allele frequency (%); SE, standard error; P, p-value; D, direction of effect; ChiSq, heterogeneity test statistic; DF, degrees of freedom for heterogeneity test; HetP, heterogeneity p-value; n.a., not available.

**Significant after multiple-testing correction ($p < 0.00167$) in the meta-analysis; *Nominal significance ($p < 0.05$) in the meta-analysis

Model (percent change in serum urate = age + sex + systolic blood pressure + body mass index + SNP) was fitted using a 2-step approach, followed by meta-analysis.

Supplementary Table 2. Associations between SNPs and percent changes in serum urate, including heterogeneity statistics from METAL (cont.)

				60 – 120 minutes																
**Nearest gene**	**SNP**	**Allele**		**Black (N = 20)**				**White (N = 37)**				**Meta-analysis**								
		**E**	**O**	**EAF**	**Beta**	**SE**	***P***	**EAF**	**Beta**	**SE**	***P***	**EAF**	**Beta**	**SE**	***P***	**D**	**I^2**	**ChiSq**	**DF**	**Het*P***
ABCG2	rs2231142	T	G	0.00	n.a	n.a	n.a	8.11	0.02	0.01	0.12	5.36	0.02	0.01	0.11	?+	0	0.00	0	1.00
SLC2A9	rs16890979	T	C	52.50	-0.01	0.01	0.20	17.57	0.00	0.01	0.92	29.82	-0.01	0.01	0.31	-+	0	0.72	1	0.40
SLC17A1	rs1183201	A	T	7.50	-0.02	0.01	0.27	41.89	0.00	0.01	0.82	29.82	-0.01	0.01	0.45	--	0	0.79	1	0.37
SLC2A9	rs737267	T	G	52.63	-0.01	0.01	0.06	20.27	0.00	0.01	0.80	31.25	-0.01	0.01	0.12	-+	40.5	1.68	1	0.19
SLC2A9	rs6449213	C	T	26.32	-0.01	0.01	0.06	14.86	0.01	0.01	0.29	18.75	-0.01	0.01	0.32	-+	75.7	4.12	1	0.04
SLC2A9	rs3775948	C	G	39.47	-0.02	0.01	0.00	18.92	0.01	0.01	0.57	25.89	-0.01	0.00	1.73E -03*	-+	82.4	5.67	1	0.02
TRIM46	rs11264341	T	C	23.68	0.01	0.01	0.18	48.65	0.01	0.01	0.29	40.18	0.01	0.01	0.09	++	0	0.17	1	0.68
INHBB	rs17050272	A	G	10.53	-0.03	0.01	0.00	51.35	-0.01	0.01	0.51	37.50	-0.02	0.01	5.92E -05**	--	78.4	4.63	1	0.03
ORC4	rs2307394	G	A	23.68	-0.01	0.01	0.49	29.73	-0.01	0.01	0.44	27.68	-0.01	0.01	0.29	--	0	0.00	1	0.98
LRRC16A	rs9358856	A	G	10.53	0.02	0.01	0.14	14.86	-0.01	0.01	0.29	13.39	0.00	0.01	0.70	+-	70.6	3.40	1	0.07
SLC17A3	rs2762353	T	C	5.26	-0.03	0.01	0.03	40.54	0.00	0.01	0.92	28.57	-0.01	0.01	0.30	-+	77.8	4.51	1	0.03
SLC17A1	rs1165151	A	C	7.89	-0.01	0.01	0.31	41.89	0.00	0.01	0.82	30.36	0.00	0.01	0.47	--	0	0.60	1	0.44
SLC22A7	rs4149178	G	A	31.58	0.00	0.01	0.91	14.86	0.00	0.01	0.94	20.54	0.00	0.01	0.95	+-	0	0.02	1	0.90
BAZ1B	rs1178977	G	A	21.05	0.00	0.01	0.73	6.76	0.02	0.02	0.23	11.61	0.00	0.01	0.80	-+	34.3	1.52	1	0.22
PRKAG2	rs10480300	T	C	36.84	0.00	0.01	0.64	17.57	0.02	0.01	0.11	24.11	0.00	0.01	0.64	-+	63.9	2.77	1	0.10
MBOAT4	rs7813902	T	C	21.05	-0.01	0.01	0.41	2.70	-0.01	0.02	0.82	8.93	-0.01	0.01	0.38	--	0	0.00	1	0.96
HNF4G	rs2941484	C	T	15.79	0.00	0.01	0.75	47.30	0.01	0.01	0.32	36.61	0.01	0.01	0.32	++	0	0.11	1	0.74
A1CF	rs10821905	A	G	31.58	0.01	0.01	0.38	16.22	0.00	0.01	1.00	21.43	0.00	0.01	0.48	++	0	0.32	1	0.57
SLC16A9	rs12356193	G	A	7.89	0.01	0.01	0.53	20.27	-0.01	0.01	0.53	16.07	0.00	0.01	0.87	+-	0	0.79	1	0.37
SLC16A9	rs1171614	A	G	23.68	0.01	0.01	0.35	25.68	0.00	0.01	0.66	25.00	0.01	0.01	0.32	++	0	0.15	1	0.69
NRXN2	rs478607	G	A	55.26	0.00	0.01	0.59	20.27	-0.01	0.01	0.50	32.14	0.00	0.01	0.97	+-	0	0.75	1	0.39
RERG	rs11056399	T	C	47.37	-0.01	0.01	0.12	36.49	0.00	0.01	0.91	40.18	-0.01	0.00	0.15	--	0	0.64	1	0.43
ACVR1B/ ACVRL1	rs7976059	T	G	18.42	0.00	0.01	0.73	39.19	0.00	0.01	0.79	32.14	0.00	0.01	0.66	--	0	0.01	1	0.95
INHBC	rs3741414	A	G	13.16	0.00	0.01	0.98	22.97	0.00	0.01	0.70	19.64	0.00	0.01	0.76	+-	0	0.07	1	0.80
B3GNT4	rs7953704	A	G	36.84	-0.01	0.01	0.31	48.65	0.00	0.01	0.77	44.64	0.00	0.01	0.69	-+	1.8	1.02	1	0.31
IGF1R	rs6598541	A	G	57.89	0.00	0.01	0.91	24.32	0.00	0.01	0.92	35.71	0.00	0.01	0.99	+-	0	0.02	1	0.88
UMOD	rs4293393	C	T	15.79	0.00	0.01	0.77	25.68	0.01	0.01	0.23	22.32	0.01	0.01	0.27	++	0	0.36	1	0.55
HLF	rs7224610	C	A	0.00	n.a	n.a	n.a	32.43	0.00	0.01	0.89	21.43	0.00	0.01	0.89	?+	0	0.00	0	1.00
QRICH2	rs164009	G	A	78.95	-0.01	0.01	0.40	28.38	-0.01	0.01	0.15	45.54	-0.01	0.01	0.09	--	0	0.10	1	0.76
INSR	rs1035942	T	C	34.21	0.01	0.01	0.20	32.43	0.00	0.01	0.85	33.04	0.00	0.01	0.48	+-	21.8	1.28	1	0.26

SNP, single nucleotide polymorphism; E, effect allele; O, other allele; EAF, effect allele frequency (%); SE, standard error; P, p-value; D, direction of effect; ChiSq, heterogeneity test statistic; DF, degrees of freedom for heterogeneity test; HetP, heterogeneity p-value; n.a., not available.

**Significant after multiple-testing correction ($p < 0.00167$) in the meta-analysis; *Nominal significance ($p < 0.05$) in the meta-analysis

Model (percent change in serum urate = age + sex + systolic blood pressure + body mass index + SNP) was fitted using a 2-step approach, followed by meta-analysis.

Supplementary Table 2. Associations between SNPs and percent changes in serum urate, including heterogeneity statistics from METAL (cont.)

				120 – 180 minutes																
**Nearest gene**	**SNP**	**Allele**		**Black (N = 20)**				**White (N = 37)**				**Meta-analysis**								
		**E**	**O**	**EAF**	**Beta**	**SE**	***P***	**EAF**	**Beta**	**SE**	***P***	**EAF**	**Beta**	**SE**	***P***	**D**	**I^2**	**ChiSq**	**DF**	**Het*P***
ABCG2	rs2231142	T	G	0.00	n.a	n.a	n.a	8.11	0.02	0.01	0.09	5.36	0.02	0.01	0.08	?+	0	0.00	0	1.00
SLC2A9	rs16890979	T	C	52.50	0.00	0.00	0.70	17.57	0.00	0.01	0.62	29.82	0.00	0.00	0.92	-+	0	0.39	1	0.53
SLC17A1	rs1183201	A	T	7.50	-0.01	0.01	0.17	41.89	0.00	0.01	0.98	29.82	0.00	0.00	0.37	--	19.5	1.24	1	0.27
SLC2A9	rs737267	T	G	52.63	0.00	0.00	0.70	20.27	0.00	0.01	0.68	31.25	0.00	0.00	0.88	-+	0	0.30	1	0.58
SLC2A9	rs6449213	C	T	26.32	0.00	0.00	0.51	14.86	0.00	0.01	0.64	18.75	0.00	0.00	0.41	++	0	0.01	1	0.94
SLC2A9	rs3775948	C	G	39.47	0.00	0.00	0.97	18.92	0.00	0.01	0.86	25.89	0.00	0.00	0.90	++	0	0.02	1	0.90
TRIM46	rs11264341	T	C	23.68	0.00	0.01	0.49	48.65	0.00	0.01	0.54	40.18	0.00	0.00	0.35	--	0	0.00	1	0.96
INHBB	rs17050272	A	G	10.53	0.00	0.01	0.50	51.35	-0.01	0.01	0.34	37.50	0.00	0.00	0.93	+-	28	1.39	1	0.24
ORC4	rs2307394	G	A	23.68	0.00	0.01	0.96	29.73	-0.01	0.01	0.23	27.68	0.00	0.00	0.46	+-	0	0.93	1	0.33
LRRC16A	rs9358856	A	G	10.53	0.01	0.01	0.03	14.86	-0.01	0.01	0.47	13.39	0.01	0.00	0.14	+-	72.1	3.59	1	0.06
SLC17A3	rs2762353	T	C	5.26	-0.02	0.01	0.04	40.54	0.00	0.01	0.89	28.57	-0.01	0.00	0.18	--	66	2.94	1	0.09
SLC17A1	rs1165151	A	C	7.89	-0.01	0.01	0.17	41.89	0.00	0.01	0.98	30.36	0.00	0.00	0.37	--	19.5	1.24	1	0.27
SLC22A7	rs4149178	G	A	31.58	0.00	0.00	0.31	14.86	0.01	0.01	0.22	20.54	0.00	0.00	0.14	++	0	0.47	1	0.49
BAZ1B	rs1178977	G	A	21.05	0.01	0.00	0.16	6.76	0.00	0.01	0.84	11.61	0.01	0.00	0.15	++	0	0.10	1	0.75
PRKAG2	rs10480300	T	C	36.84	0.00	0.00	0.31	17.57	0.02	0.01	0.05	24.11	0.00	0.00	0.95	-+	81.1	5.30	1	0.02
MBOAT4	rs7813902	T	C	21.05	0.00	0.00	0.76	2.70	0.01	0.02	0.75	8.93	0.00	0.00	0.71	++	0	0.05	1	0.82
HNF4G	rs2941484	C	T	15.79	0.01	0.01	0.18	47.30	-0.01	0.01	0.32	36.61	0.00	0.00	0.77	+-	65.3	2.88	1	0.09
A1CF	rs10821905	A	G	31.58	0.00	0.00	0.27	16.22	0.01	0.01	0.10	21.43	0.01	0.00	0.06	++	0	0.66	1	0.42
SLC16A9	rs12356193	G	A	7.89	0.00	0.01	0.84	20.27	0.00	0.01	0.96	16.07	0.00	0.01	0.86	++	0	0.01	1	0.91
SLC16A9	rs1171614	A	G	23.68	0.00	0.01	0.44	25.68	0.00	0.01	0.96	25.00	0.00	0.00	0.52	--	0	0.21	1	0.64
NRXN2	rs478607	G	A	55.26	0.00	0.00	0.59	20.27	0.00	0.01	0.99	32.14	0.00	0.00	0.64	-+	0	0.08	1	0.78
RERG	rs11056399	T	C	47.37	0.00	0.00	0.91	36.49	0.00	0.01	0.82	40.18	0.00	0.00	0.99	-+	0	0.07	1	0.79
ACVR1B/ ACVRL1	rs7976059	T	G	18.42	0.00	0.00	0.96	39.19	0.01	0.01	0.07	32.14	0.00	0.00	0.21	++	46.1	1.86	1	0.17
INHBC	rs3741414	A	G	13.16	0.00	0.01	0.81	22.97	-0.01	0.01	0.11	19.64	0.00	0.00	0.29	+-	39.7	1.66	1	0.20
B3GNT4	rs7953704	A	G	36.84	0.00	0.01	0.87	48.65	0.00	0.01	0.84	44.64	0.00	0.00	0.79	--	0	0.00	1	0.99
IGF1R	rs6598541	A	G	57.89	0.00	0.00	0.97	24.32	0.00	0.01	0.43	35.71	0.00	0.00	0.63	++	0	0.41	1	0.52
UMOD	rs4293393	C	T	15.79	0.00	0.00	0.68	25.68	0.00	0.01	0.63	22.32	0.00	0.00	0.53	++	0	0.01	1	0.92
HLF	rs7224610	C	A	0.00	n.a	n.a	n.a	32.43	0.01	0.01	0.22	21.43	0.01	0.01	0.21	?+	0	0.00	0	1.00
QRICH2	rs164009	G	A	78.95	0.01	0.01	0.13	28.38	0.00	0.01	0.84	45.54	0.00	0.00	0.28	+-	26.5	1.36	1	0.24
INSR	rs1035942	T	C	34.21	0.00	0.00	0.81	32.43	0.00	0.01	0.34	33.04	0.00	0.00	0.62	+-	0	0.76	1	0.38

SNP, single nucleotide polymorphism; E, effect allele; O, other allele; EAF, effect allele frequency (%); SE, standard error; P, p-value; D, direction of effect; ChiSq, heterogeneity test statistic; DF, degrees of freedom for heterogeneity test; HetP, heterogeneity p-value; n.a., not available.

**Significant after multiple-testing correction ($p < 0.00167$) in the meta-analysis; *Nominal significance ($p < 0.05$) in the meta-analysis

Model (percent change in serum urate = age + sex + systolic blood pressure + body mass index + SNP) was fitted using a 2-step approach, followed by meta-analysis.
